# Kinetic analysis of pathogenicity and tissue tropism of gyrovirus 3 in experimentally infected chickens

**DOI:** 10.1186/s13567-021-00990-2

**Published:** 2021-09-15

**Authors:** Gen Li, Defang Zhou, Manda Zhao, Qing Liu, Xiaojing Hao, Tianxing Yan, Shiyu Yuan, Shicheng Zhang, Ziqiang Cheng

**Affiliations:** 1 College of Veterinary Medicine, Shandong Agricultural University, Tai’an, 271018 China; 2 College of Veterinary Medicine, Qingdao Agricultural University, Qingdao, China; 3 Department of Animal Science and Technology, Vocational-Technical School of Husbandry and Veterinary Medicine, Weifang, China; 4 Service Center of Jinan Zoo, Jinan, China; 5 Qindao Husbandry and Veterinary Institute, Qingdao, China

**Keywords:** Gyrovirus 3, pathogenicity, tissue tropism, SPF chicken

## Abstract

Gyrovirus 3 (GyV3), the third novel emerging species of the genus *Gyrovirus* of the *Anelloviridae* family, has been described in multiple hosts. Epidemiologically, there are suggestions that GyV3 is associated with diarrhea/proventriculitis, however, no direct causal evidence exists between GyV3 infection and specific clinical diseases. Herein, we infected special pathogen-free (SPF) chickens with GyV3, and then assessed the pathogenicity and tissue tropism. The results revealed that GyV3 induced persistent infection characterized by diarrhea, aplastic anemia, immunosuppression, and persistent systemic lymphocytic inflammation. Clinically, the infected chickens presented ruffled feathers, diarrhea, anemia, and weight loss. Aplastic anemia was characterized by progressive depletion of hematopoietic cells in the bone marrow, immunosuppression was associated with atrophy of the thymus, spleen, and bursa of Fabricious, progressive lymphocytic inflammations were characterized by proventriculitis, adrenalitis, pancreatitis, hepatitis, nephritis, and bronchitis. Viral loads of GyV3 in tissues exhibited “M”, “N”, “W” or “V” type dynamic changes. The highest level of viral loads was reported in bone marrow at 7dpi, followed by the adrenal gland at 2 dpi, the sciatic nerve at 7 dpi, and bile at 35 dpi. The bone marrow and kidney demonstrate the strongest immunostaining of GyV3-VP1 antigen and were suggested as the target tissues of GyV3. Collectively, GyV3 is an immunosuppressive pathogenic virus that targets the bone marrow and kidney in chickens. Exploring the pathogenicity and tissue tropism of GyV3 will guide the basic understanding of the biology of GyV3 and its pathogenesis in chickens.

## Introduction

The genus *Gyrovirus* (GyV) of the *Anelloviridae* family, comprises a group of small, non-enveloped, icosahedral viruses with a circular single-stranded approximately 2.3-kb long DNA (ssDNA) genome (negative-sense). The chicken anemia virus (CAV) prototype and thirteen other unofficially recognized species (HGyV/AGV2, GyV3 through GyV13) belongs to the GyV genus that have been described in humans, ferrets, cats, wild birds, sea birds, chickens, pigeons, and ducks [1–12]. Originally, the genus Gyrovirus was classified in the Circoviridae family but later reassigned to the *Anelloviridae* family in 2017 by the International Committee on Taxonomy of Virus (ICTV) who argued that its structure or genetics was unrelated to the members of the *Circoviridae* family [13].

Gyrovirus 3 (GyV3) genome is 2356 bp long and encapsidated into 26–28 nm in diameter virions. GyV3 was identified for the first time in 2012 in faeces of Chilean and Hong Kong children with acute gastroenteritis [3]. Consequently, Chu et al. [4] found that GyV3 exhibited a 5.8% positive rate in 191 faecal specimens sampled in 2011 from patients with diarrhea. Fehér et al. [14] investigated human gyroviruses in Hungary pet ferrets with a background disease characterized by a lymph node, spleen enlargement and diarrhea, whereby GyV3 demonstrated a 20% (2/20) infection rate. In the same year, GyV3 was detected in stools from healthy children (17/49, 34.7%) and patients with diarrhea (22/149, 14.8%) in South Africa [15], and in chicken meat from stores in San Francisco [6]. A new genotype of GyV3 was again detected by Fehér et al. [16] in a fecal sample of a pet ferret. We also reported GyV3 in broiler chickens with proventriculitis in our laboratory, which exhibited a 12.5% infection rate [17]. Recently in 2019, GyV3 was found in a fecal sample of one normal cat and two diarrhetic cats from 197 fecal samples collected from pet cats in northeast China [18]. Based on the sequence analysis of cat-sourced GyV3, the authors deduced that the virus potentially originated from GyV3 infected chickens.

Currently, no cell culture system has been established for GyV3 propagation, and an animal model that can provide information on the virus-host interactions is lacking. Here, we constructed a reproductive infection model of GyV3 broiler-origin for specific pathogen-free (SPF) chickens and evaluated the pathogenicity and tissue tropism of GyV3.

## Materials and methods

### Virus and animals

GyV3-SDAU-1 strain (GenBank accession no. MG366592, identified from broiler chicken) was stored in our Molecular Pathology Laboratory. Based on previous reports, we isolated GyV3 from the kidney. Briefly, kidneys from GyV3 positive chicks were homogenated in DMEM (1 g kidney + 5 mL DMEM) with 30 μL penicillin (3000 IU/mL) and 30 μL streptomycin (3 mg/mL). The homogenate was placed in an ice bath. Then, using an ultrasonic breaker, the tissue was crushed for 3 s at intervals of 3 s, 99 × 3 times, differential centrifugation at 4 °C (9000 × *g*/min, 10 min and 11 000 × *g*/min,10 min). The supernatant collected after differential centrifugation was passed through a 0.22 μm filter. We inoculated the supernatant into a blood agar plate and nutrient broth plate and incubated it at 37 °C overnight. Bacterial negative viral fluid was used to infect chicks [1-day old SPF chicks purchased from Poultry Research Institute, Shandong Academy of Agricultural Sciences (Jinan city, China)].

### GyV3 polyclonal antibody preparation

An anti-GyV3 polyclonal antibody was prepared from rabbits. Briefly, recombinant fusion proteins of pET32a-GyV3-VP1 were expressed in *Escherichia coli* (Tiangen Biotech, Beijing, China). Rabbits were immunized with the protein three times to obtain antiserum. Then, we used the purified antiserum as the primary antibody for immunohistochemistry (IHC). Serum collected from VP1-free rabbits served as the negative control.

### Experimental design

Sixty 1-day-old SPF chicks were categorized into the control and GyV3 infected groups. On day 1, each chick of the infection group was intraperitoneally infected with 200 µL of 3.37 × 10^7^ viral copies of the GyV3-SDAU-1 strain quantified via quantitative real-time PCR (qPCR) from the supernatant of kidney homogenate of the GyV3 infected SPF chicks. The weights and deaths of the birds were recorded daily. Sera were used to detect the viral infection rate through PCR 7 days post-infection (dpi). Three chickens were euthanized and necropsied at 2, 3, 5, 7, 14, 21, 28, and 35 dpi, respectively. In the control group, three animals were euthanized and necropsied at the same duration intervals, respectively. We collected 29 tissues from feather pulp, skin, pancreas, ovary or testis, footpad, trachea, lung, bone marrow, spleen, thymus, bursa of Fabricius, heart, pectoralis, crureus, kidney, adrenal gland, cerebrum, cerebellum, sciatic nerve, liver, bile, proventriculus, ventriculus, duodenum, jejunum, ileum, cecum, colon and rectum for histopathological examination, qPCR and IHC assay.

### Histopathology

Upon necropsy, tissues were fixed in 10% neutral formalin for 48 h at room temperature, embedded in paraffin, and cut into 4-μm-thick sections. After deparaffinization, the sections were subjected to hematoxylin and eosin (H&E) staining and examined using a light microscope (Olympus, Tokyo, Japan).

### Quantitative real-time PCR (qPCR)

Viral DNA copy number from tissues of GyV3 infected chickens was established through extrapolation from a GyV3 plasmid-based standard curve. We applied the DNASTAR software (DNASTAR Inc., Madison, WI, USA) to verify the highly conserved regions in the GyV3-SDAU-1 genomes. Primers were designed using the Primer 5.0 software (Applied Biosystems, Foster City, CA, USA). The following primer pair was used: Forward, GyV3-F: 5′-ACCGGGACTTGGACACC-3′, reverse, GyV3-R: 5′-AGCCAGGAAGCGATACG-3′. The GyV3 amplicons were 155 base pairs in length. The PCR products were cloned into the pMD18-T vector. The constructed plasmids pMD18-T were transformed into DH5α competent cells (Beijing QIAGEN Biochemical Technology Co., Ltd, China) and then extracted using QIAGEN Plasmid Midi Kits. The plasmids served as templates for real-time PCR optimization. An OSE260 spectrophotometer (NanoDrop, Beijing, China) was used to quantify the plasmids. The copy numbers of the plasmids were calculated as follows: log10 copies/μL = 6.02 × 1023 (copies/mol) × plasmid concentration (ng/μL) × 10^–9^/[(molecular weight of vector + molecular weight of inserted fragment) × 660] (g/mol)). To test the sensitivity of the established real-time PCR, the constructed plasmids were diluted from 10^9^ to 10^3^ in TE buffer (10 mmol/L Tris–HCl, 1 mmol/L EDTA), and then used as templates to generate a standard curve for GyV3. The threshold cycle (Ct) of these standard dilutions was plotted against the log value of the copy number of the corresponding standard plasmid. Real-time PCR was performed using a Roche LightCycler96 machine (Roche Ltd, Switzerland). The real-time PCR mixture comprised 10 μL TB Green^®^ *Premix Ex Taq*™ (TaKaRa Bio, Dalian, China), 0.4 μL forward primer (10 μmol/L), 0.4 μL reverse primer (10 μmol/L), 1 μL template DNA, and sterile water to attain a final volume of 20 μL. The amplification parameters were set as follows: 95 °C for 30 s, followed by 40 cycles of 95 °C for 5 s, and 60 °C for 30 s, 95 °C 1 s, 60 °C 15 s, 95 °C 1 s. Roche fluorescence quantitative PCR system automatically generated a standard curve.

### Immunohistochemistry (IHC)

Serial sections (4 µm) of selected tissues from chickens were immunolabelled for GyV3 VP1 antigen using polyclonal antibodies specific for VP1 protein following a routine process. The labeling intensity and the number of immuno-labeled GyV3-positive cells in the tissues were recorded as previously described by Castaño et al. [19].

### Statistical analysis

All statistical data were analyzed in Graphpad Prism software, version 7.00. A Student *t*-test was applied to analyze the differences in mean values between groups. A *P* value < 0.05 denoted statistical significance. (*P values of ≤ 0.05. ** values of ≤ 0.01. ***P values of ≤ 0.001.) All values are presented as mean ± SEM.

## Results

### Clinical characteristics

**Figure 1:**
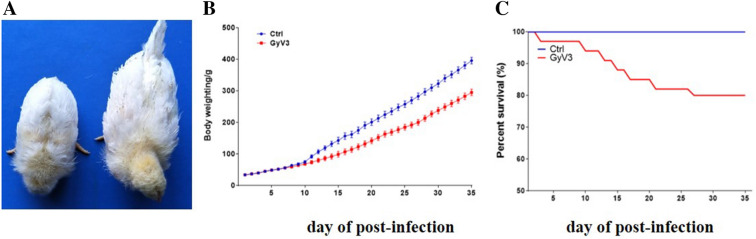
**Clinical characteristics. A** Clinical symptoms showing ruffled feathers, diarrhea, anemia, and weight loss. **B** The weight gain of GyV3 infected chickens is significantly lower than control chickens from 10 dpi. **C** Survival rate.

Chickens infected with GyV3 developed an acute course of disease with 90% (27/30) morbidity. They presented ruffled feathers, diarrhea and anemia (Figure 1A), and significant weight loss (from 10 dpi onwards) (Figure 1B). The total mortality was 20% (6/30) in GyV3 infected chicks. Death occurred at 7 dpi (1 chicken), 21 dpi (2 chickens) and 28 dpi (1 chicken) (Figure 1C). The clinical symptoms of most infected chickens were alleviated from 35 dpi. All chicks in the control group tested negative for GyV3 antibodies throughout the experiment.

### Pathogenicity of GyV3

**Figure 2:**
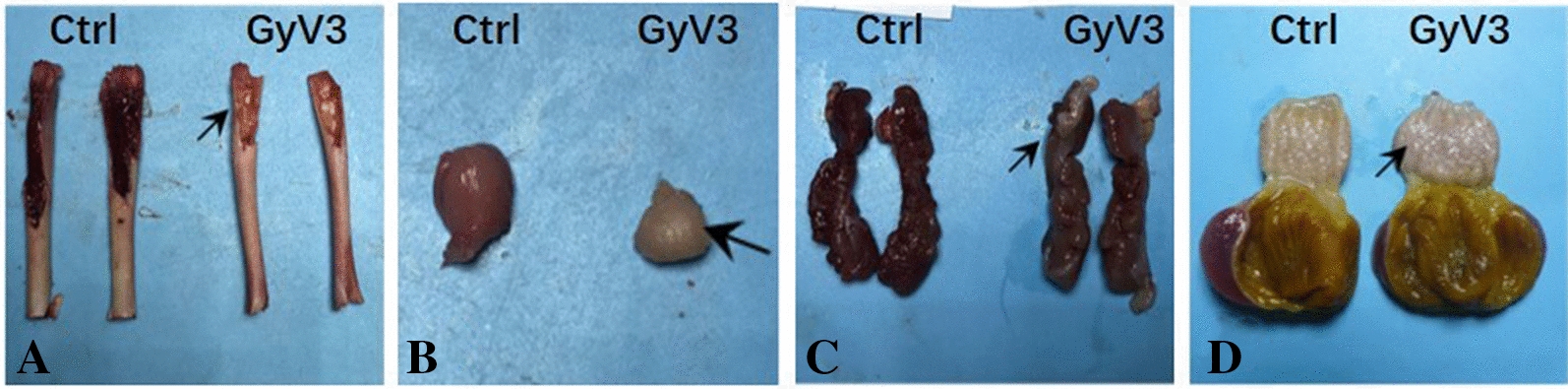
**Gross lesions caused by GyV3. A** Yellowish-white bone marrow. **B** Atrophied bursa of Fabricius. **C** Pale kidney. **D** Swollen proventriculus.

**Figure 3:**
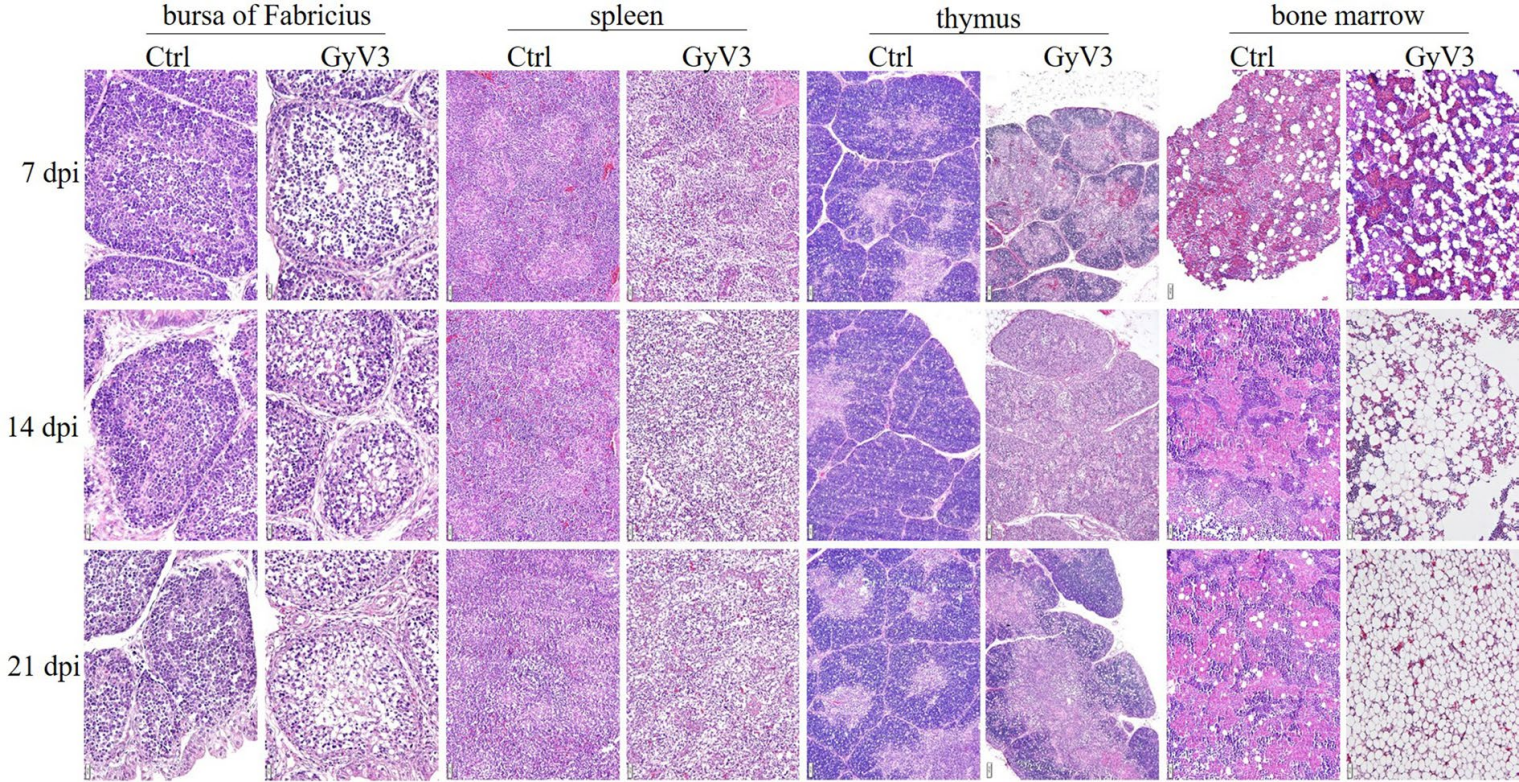
**Histopathology lesions of immune organs induced by GyV3 infection.** Progressive lymphocyte depletion in the bursa of Fabricius, spleen, and thymus. Hematopoietic cells were progressively replaced by adipocytes in bone marrow (HE, 10×).

**Figure 4:**
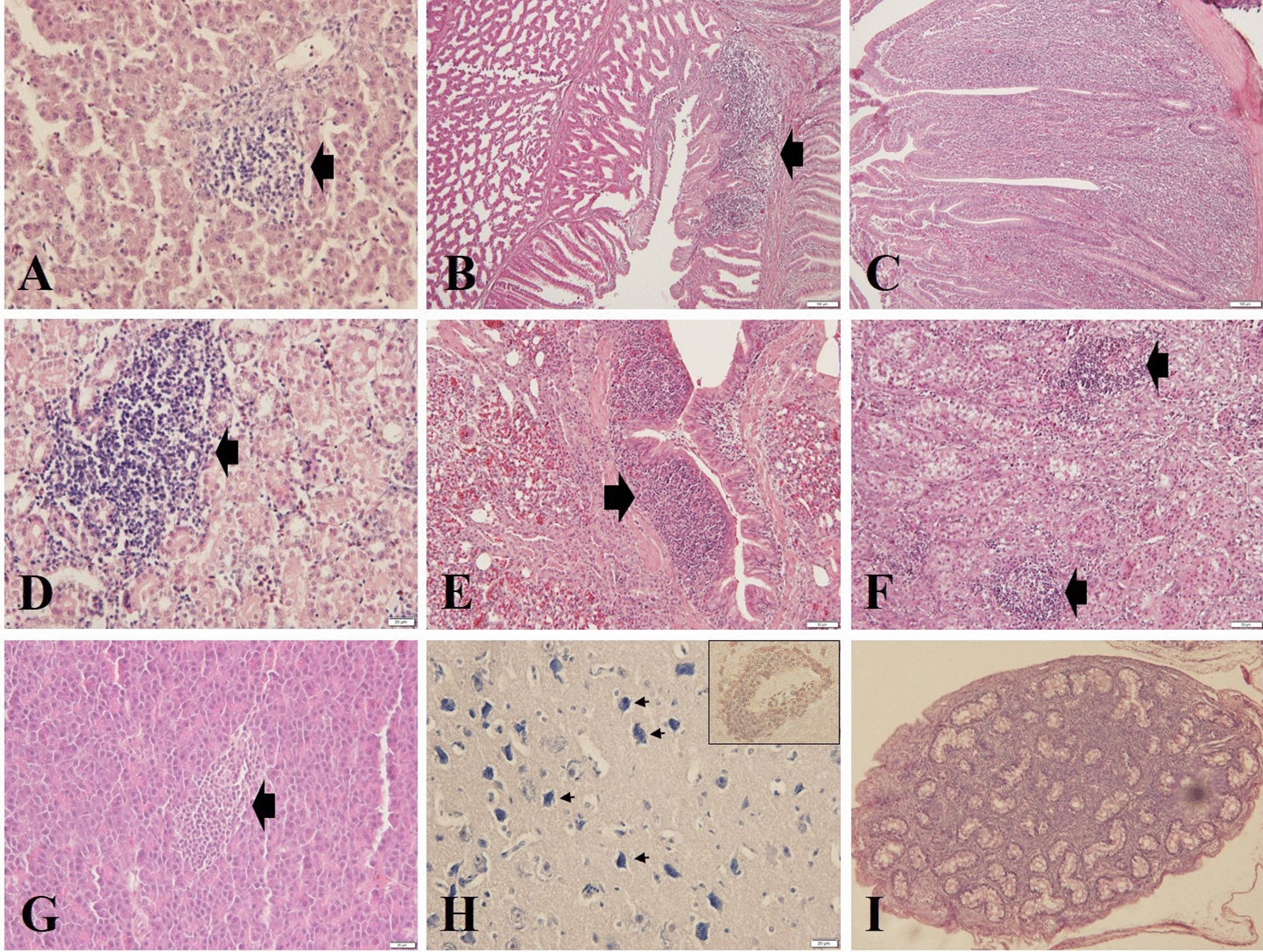
**Systematic lymphocytic inflammation and proliferation caused by GyV3 (HE). A** Hepatitis (20×). **B** Proventriculitis (10×). **C** Enteritis (10×). **D** Nephritis (20×). **E** Bronchitis (10×). **F** Adrenalitis (20×). **G** Pancreatitis (20×). **H** Inclusion bodies in the nucleus of neurons and encephalitis (small frame) (20×). **I** Interstitial proliferation in the testicle (10×).

GyV3 infected chickens presented with progressive immunosuppression, aplastic anemia, and systematic lymphocytic inflammation from 7 dpi. The gross lesions reported in GyV3 infected birds were characterized by yellowish-white bone marrow (Figure 2A), atrophied bursa of Fabricius (Figure 2B), light-colored kidney (Figure 2C), and swollen proventriculus (Figure 2D). Histopathological examination revealed immunosuppressive lesions characterized by severe lymphoid depletion with increased medullary spaces and reticular hyperplasia in the bursa of Fabricius, spleen, and thymus of the infected birds. Aplastic anemia was characterized by hematopoietic cells, progressively replaced by adipocytes in the bone marrow (Figure 3). Besides, systematic lymphocytic inflammation was associated with moderate to severe hepatitis (Figure 4A), proventriculitis (Figure 4B), enteritis (Figure 4C), nephritis (Figure 4D), bronchitis (Figure 4E), adrenalitis (lymphoplasmacytic inflammation that induces more destruction of adrenocortical tissue) (Figure 4F), pancreatitis (Figure 4G) and encephalitis (Figure 4H, small frame). Injury lesions, including edema, degeneration, necrosis, or proliferation were found in all inflammatory tissues. Inclusion bodies were present in the nucleus of neurons in the cerebrum (Figure 4H), whereas interstitial proliferation occurred in the testicle (Figure 4I). The onset of the lesions was at 7 dpi. Severe characteristic lesions developed on 14 and 21 dpi. For most infected chickens, the lesions were alleviated from 28 dpi. All chickens in the uninfected control groups remained healthy throughout the experimental period without any signs or lesions.

### Replication kinetics of GyV3 in tissues

**Figure 5:**
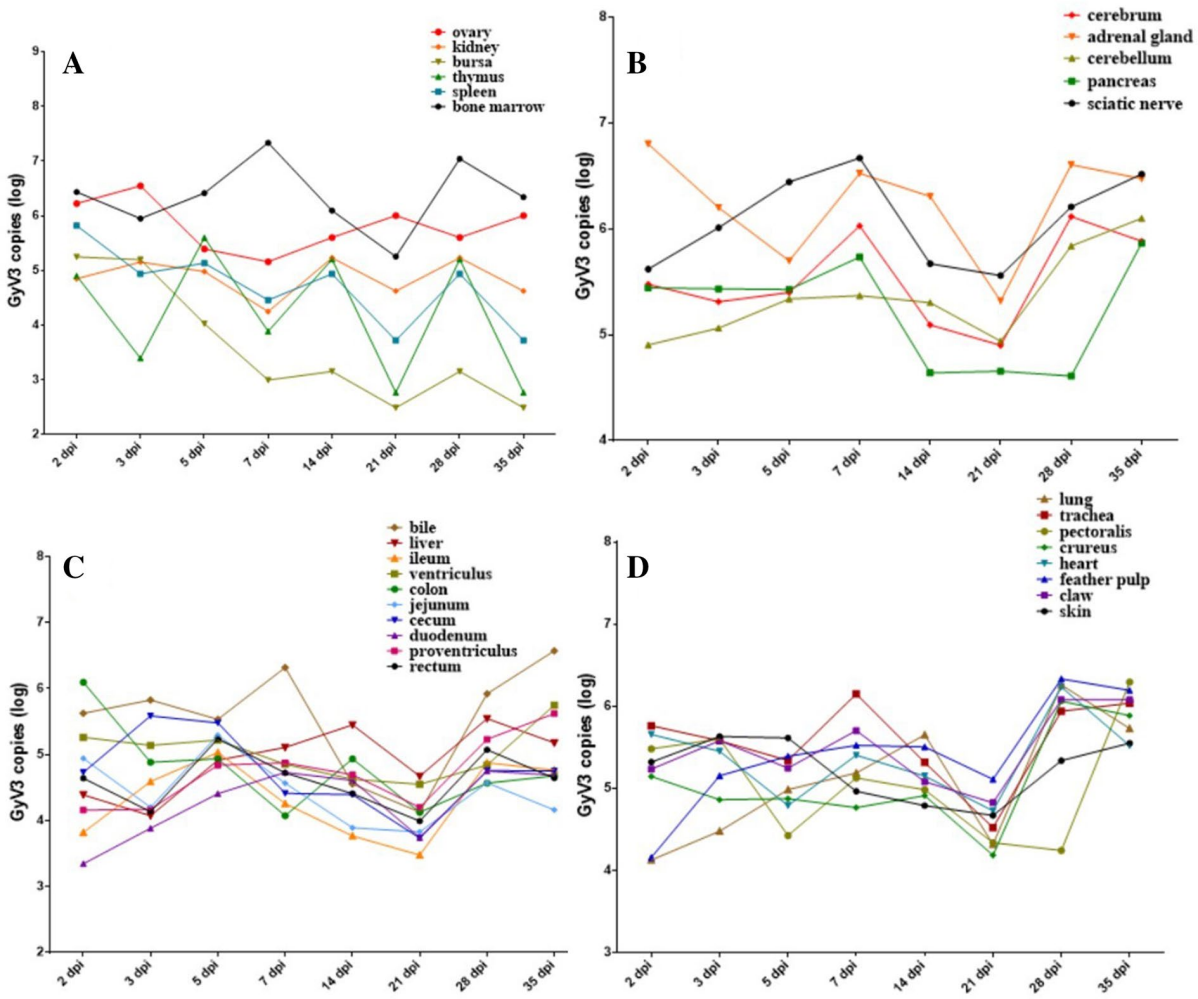
**Kinetic of GyV3 loads in tissues.** The copies of GyV3 DNA in 29 tissues, including **A** immune and urogenital systems. **B** neuroendocrine system. **C** digestive system, and **D** other systems were estimated employing absolute qPCR.

Using qPCR, copies of the GyV3 genome were quantified in 29 tissues of infected chickens on 2, 3, 5, 7, 14, 21, 28, and 35 dpi. Figure 5 shows active replication of GyV3 detected in all 29 tissues throughout the experimental period. Its kinetic replication in tissues showed “M”, “N”, “W” or “V” type fluctuating trend. “M” type viral loads of GyV3 were detected in tissues of the bone marrow, cerebrum, liver, lung, kidney, “N” type was reported in the sciatic nerve, bile, ileum, duodenum, proventriculus, “W” type was found in the ovary, thymus, spleen, adrenal gland, colon, jejunum, cecum, rectum, trachea, pectoralis, heart, and “V” type was found in the pancreas, crureus, claw, skin. Additionally, the viral loads of GyV3 in the bursa of Fabricius and ventriculus continuously decreased, whereas that in feather pulp continuously increased. The highest viral load level was reported in bone marrow at 7 dpi (7.33 ± 1.12 log_10_ copies/µL), followed by an adrenal gland at 2 dpi (6.80 ± 0.08 log_10_ copies/µL) and bile at 35 dpi (6.57 ± 0.28 log_10_ copies/µL). The lowest viral loads in nearly all tissues were reported on 21 dpi, which implicated 21 dpi as the critical point of GyV3 infection.

### GyV3 tissue tropism

**Figure 6:**
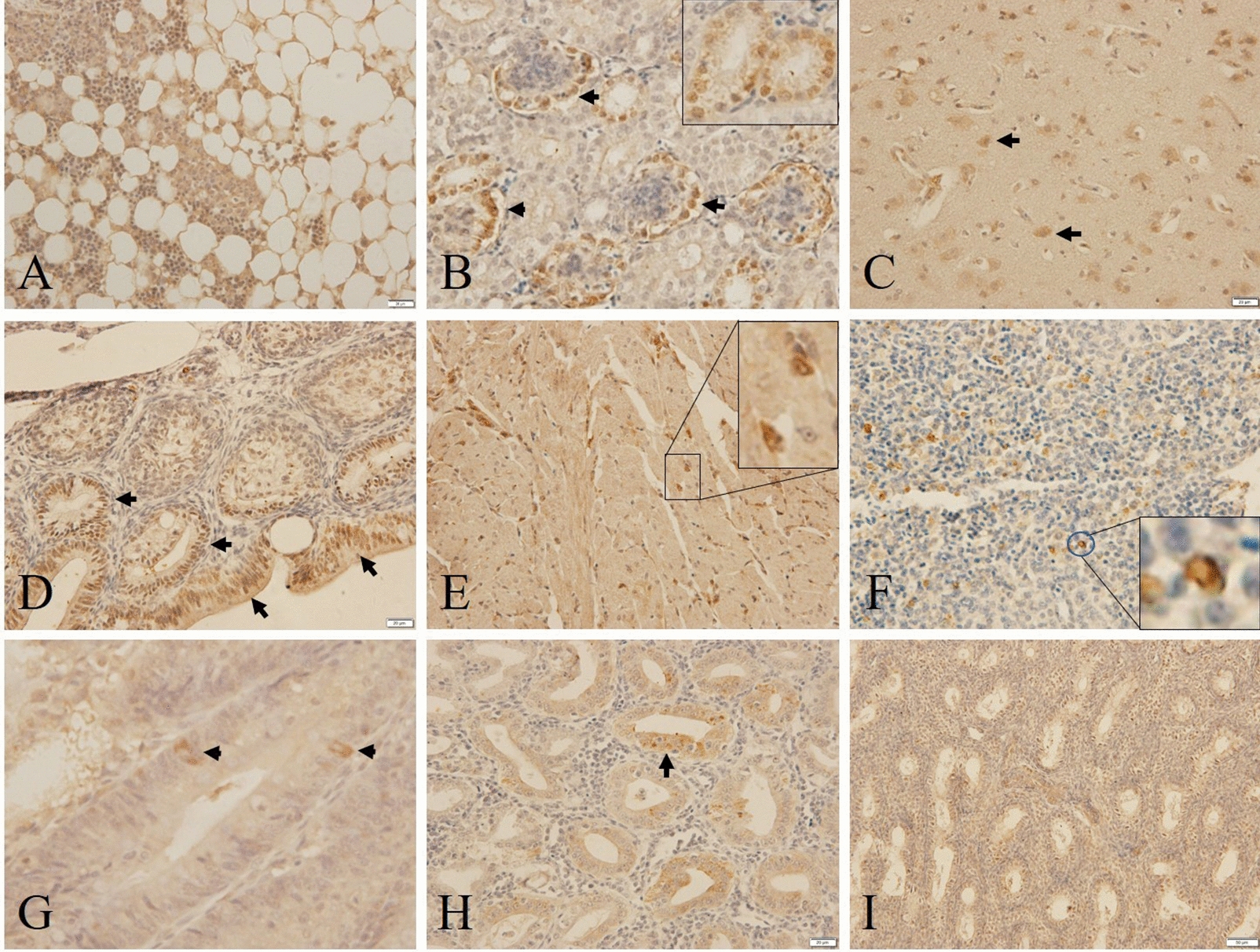
**Distribution and localization of GyV3-VP1 (IHC). A** Strong immunostaining present in myeloid cells in bone marrow. **B** Epithelial cells in the nephric tubule, ureter, glomerulus of kidney. **C** Neurons in the cortex of the cerebrum, and **D** epithelial cells and lymphocytes in the bursa of Fabricius. Intermediate immunostaining present in **E** myocardial cells in heart. **F** Lymphocytes of in spleen. **G**, **H** Epithelia in villus epithelia and glandular epithelia in proventriculus and intestine. **I** Spermatogenic cell located in tubuli seminiferi contorti in the testicle.

To understand the tissue tropism and target organs of GyV3, the distribution and localization of GyV3-VP1 were evaluated via IHC in various tissues at 27 dpi. GyV3 displayed restricted tissue tropism. The strongest immunostaining was detected in myeloid cells of bone marrow (Figure 6A), epithelial cells in the nephric tubule, ureter, glomerulus of the kidney (Figure 6B), neurons in the cortex of cerebrum (Figure 6C), and lymphocytes and epithelial cells in the bursa of Fabricius (Figure 6D). The scattered positive immunostaining was revealed in myocardial cells of the heart (Figure 6E), lymphocytes of the spleen (Figure 6F), epithelial cells in villus epithelia and glandular epithelia in proventriculus and intestine (Figures 6G and H), bronchus epithelial cells in the lung, and spermatogenic cells localized in tubuli seminiferi contorti in the testicle (Figure 6I).

## Discussion

Large-scale and highly diverse novel Anelloviruses have presently been identified through viral metagenomics of different hosts or environments [20]. However, most of them are yet to be associated with any disease. Since 2011, 13 GyV species of the *Gyrovirus* genus of the *Anelloviridae* family have been reported in multiple natural hosts. Compelling evidence shows that several GyV are associated with gastroenteritis or neurological signs but their potential pathogenicity remains elusive. Previously, we identified GyV3 from commercial broiler chickens with TVP and confirmed its epidemic in chicken flocks [17]. In the present study, we established an infection model of GyV3 in SPF chickens to explore the pathogenicity and tissue tropism.

As for the source of GyV3, Niu et al. [18] had speculated that diverse cat-origin gyroviruses potentially originated from gyrovirus-infected chickens or humans harboring gyroviruses via the fecal–oral route, and domestic cats were the unlikely natural host for gyroviruses. Based on the adaptability in SPF chickens, we speculate that commercial broiler chickens may be the original host of GyV3. The rapid adaptation of GyV3 in layer chickens is likely to cause a pandemic in the layer poultry industry in the future.

Infections by viruses of the *Anelloviridae* family are in most cases, asymptomatic [21], except for a member of genus *Gyrovirus*, chicken anemia virus (CAV), which potentially can cause illness in young chickens [22]. Like CAV, clinical signs and gross lesions implicate GyV3 as an immunosuppressive pathogenic virus characterized by stunted growth, aplastic anemia, and immune organ atrophy. Histopathological examination revealed that GyV3 could cause persistent systematic infection, particularly in the immune system (bone marrow failure, atrophy of bursa of Fabricious, spleen, and thymus), endocrine system (adrenalitis and pancreatitis), digestive system (hepatitis, proventriculitis, and enteritis) and urinary system (nephritis), which might be directly associated with acute death of GyV3 infected chickens.

Several specific lesions induced by GyV3 should be of high concern. We reported a majority of bone marrow failures characterized by aplastic anemia during GyV3 infection. The high GyV3 loads and strong VP1 immunolabelling further confirmed that the bone marrow is a potential direct target for GyV3, inducing aplastic anemia. Viral metagenomics revealed 46 different species of Anelloviruses in bone marrow specimens from hematologic patients [23], suggesting bone marrow as one of the target organs. GyV3 infection was also linked to immunosuppression, characterized by severe depletion of lymphocytes in the thymic cortex, lymphoid areas in the spleen, and depletion in the bursa of Fabricius. These changes demonstrated that the virus primarily attacks the lymphoid tissues. The typical characteristics of aplastic anemia and immunosuppression were previously revealed in other immunosuppressive viruses, including CAV [24], equine infectious anemia virus (EIAV) [25], retroviruses [26], circoviruses [27, 28], parvovirus B19, Epstein–Barr virus (EBV), cytomegalovirus (CMV), varicella-zoster virus (VZV), human herpes virus, dengue virus [29], and so on. However, the mechanism by which these viruses induce aplastic anemia and immunosuppression is yet to be fully explored.

Besides, the high neuroendocrine-tropism of GyV3 is an apathogenic feature. We found that high-level GyV3 loads or VP1 expression in the cerebellum, sciatic nerve, cerebellum, adrenal gland, and pancreas were associated with viral encephalitis, adrenalitis, and pancreatitis. Previously, in genus *Gyroviruses*, GyV8 was described in a case of ataxia in a northern fulmar [7], however, the causative association between GyV8 and the ataxia could not be established. Elsewhere, GyV10 has been identified in the species *Chauna torquata*, associated with a neurological disease with clinical and pathological characteristics [9]. Other scholars had detected Torque teno virus (TTV) microRNA in cerebrospinal fluids of patients with neurological pathologies [30, 31]. Therefore, neurotropism may be the common characteristic of Gyroviruses, nevertheless, endocrine-tropism is yet to be reported in Gyroviruses.

Detection of viral loads and GyV3-VP1 antigen expression revealed that GyV3 exhibits a persistent active replication and broad tropism in body tissues. These events contribute to the clinical manifestations and epidemiology observed in the current epidemic. As such, most researchers believed that fecal–oral is the transmission route of Gyroviruses. In our study, early and late stages of infection, and high GyV3 loads were reported in the ovary, trachea, and feather pulp, respectively. We concluded that GyV3 can efficiently spread via vertical and horizontal transmission routes. In addition, we found no significant correlation between the severe gastroenteritis and the low viral load of the digestive canal, an implication that local GyV3 replication in the digestive canal occurred in a few target cells. Thus, further exploration is warranted to identify the concrete target cells. The timing of peak viral loads (1 dpi) did not coincide with the timing of peak disease severity (21 dpi). Conversely, at the lowest point, 21 dpi, GyV3 infected chickens were characterized by severe lesions. Therefore, the impact of GyV3 load on histopathological changes should be further explored.

The present study implicates GyV3 as an immunosuppressive pathogenic virus with bone marrow as the principal target organ that mediates the immunosuppression. The basic understanding of the pathogenicity and tissue tropism of GyV3 will be vital in the understanding of the biology of GyV3, and reveal the pathogenesis in chickens.
